# Classification Method of Uniform Circular Array Radar Ground Clutter Data Based on Chaotic Genetic Algorithm

**DOI:** 10.3390/s21134596

**Published:** 2021-07-05

**Authors:** Bin Yang, Mo Huang, Yao Xie, Changyuan Wang, Yingjiao Rong, Huihui Huang, Tao Duan

**Affiliations:** 1 Institute of Microelectronics of Chinese Academy of Sciences, Beijing 100029, China; yangbin17@mails.ucas.ac.cn (B.Y.); huangmo@ime.ac.cn (M.H.); xieyao@ime.ac.cn (Y.X.); wangchangyuan@ime.ac.cn (C.W.); huanghuihui@ime.ac.cn (H.H.); 2 University of Chinese Academy of Sciences, Beijing 100049, China; 3 Science and Technology on Near Surface Detection Laboratory, Wuxi 214035, China; enjoy_rong@163.com

**Keywords:** UCA radar, ground clutter, chaotic genetic algorithm, clustering process, characteristic factor

## Abstract

The classification and recognition of radar clutter is helpful to improve the efficiency of radar signal processing and target detection. In order to realize the effective classification of uniform circular array (UCA) radar clutter data, a classification method of ground clutter data based on the chaotic genetic algorithm is proposed. In this paper, the characteristics of UCA radar ground clutter data are studied, and then the statistical characteristic factors of correlation, non-stationery and range-Doppler maps are extracted, which can be used to classify ground clutter data. Based on the clustering analysis, results of characteristic factors of radar clutter data under different wave-controlled modes in multiple scenarios, we can see: in radar clutter clustering of different scenes, the chaotic genetic algorithm can save 34.61% of clustering time and improve the classification accuracy by 42.82% compared with the standard genetic algorithm. In radar clutter clustering of different wave-controlled modes, the timeliness and accuracy of the chaotic genetic algorithm are improved by 42.69% and 20.79%, respectively, compared to standard genetic algorithm clustering. The clustering experiment results show that the chaotic genetic algorithm can effectively classify UCA radar’s ground clutter data.

## 1. Introduction

Uniform circular array (UVA) radar is a uniform circular array radar composed of isotropic antennas, which has the advantages of space omnidirectional scanning, various search and tracking methods, and flexible beam pointing [1,2,3]. It is especially suitable for the monitoring of low and slow targets under complex terrain background. The signal received by UCA ground-based radar includes the electromagnetic scattering signal generated by the surrounding environment besides the target signal. Ground clutter refers to the echo signal formed after the electromagnetic wave signal emitted by radar is reflected by ground object background [4], which is usually affected by radar system parameters, ground object background type, wind speed and other factors. UCA ground-based radar is easily affected by strong scattering clutter signals during search and tracking missions, which is a crucial factor restricting the performance of UCA radar.

The performance of radar target detection depends on recognizing clutter characteristics and the estimation of relevant statistical parameters to a certain extent. Research on radar clutter signal characteristics can be summarized into four aspects: power spectrum characteristics, non-stationarity and nonlinearity, amplitude distribution characteristics, and correlation. The spectral characteristics of clutter are studied based on measured data, including basic characteristics of clutter spectrum, average spectrum shape modeling, and short-term local spectrum analysis (short-term non-stationarity and spatial non-uniformity) [5,6,7]. When the radar works at high resolution and low grazing angle, the clutter signal exhibits non-stationary characteristics [8]. The non-stationary factors research is mainly based on the analysis theory of non-stationary signals including the signal decomposition method, transform domain method, non-stationary model modeling, and other methods [9,10]. The nonlinear characteristics of clutter can be studied based on chaos theory, and then it goes through the stages of chaotic characteristics, fractal, multifractal characteristics, and neural networks [11,12,13]. Clutter data are usually stochastic and can be described in the form of statistical distribution, such as traditional amplitude distribution model [14,15], compound Gaussian distribution model [16,17], spherical invariant random process model [18], compound structure distribution model [19], etc. The correlation of clutter can reflect the degree of linear dependence between the clutter of different pulses or different spatial positions, which is generally divided into temporal correlation and spatial correlation [20,21]. The above studies on the cognition of miscellaneous characteristics can be used as the basis for extracting miscellaneous features.

Extracting the high-dimensional time-frequency features of clutter is beneficial to the formation of the high-dimensional feature space which is nonlinear separable between clutter and target [22]. The autocorrelation matrix of the radar clutter element is selected as the critical feature of clutter classification and recognition. Then, the clutter data can be effectively classified based on the unsupervised clustering method [23]. The method of clutter classification and recognition is based on the difference of amplitude modulation characteristics of different echo signals in the range-Doppler domain [24,25], and the disadvantage of this method is that it requires a relatively high signal-to-noise ratio. In a practical case, the radar system parameters and application scenarios should be considered to determine the clutter classification characteristics to be extracted. For different clutter environments, the optimal radar signal processing and detection strategies are different [26,27,28]. In order to realize the adaptive matching of the radar signal processor to the working environment, it is necessary to classify and recognize the radar clutter in different environments. At present, the methods of radar clutter identification and classification mainly include artificial classification, spectrum analysis, autoregressive model, amplitude distribution analysis, parametric and non-parametric statistical determination, and machine learning methods, etc. A complete clutter classification system was constructed based on feature extraction, and the accuracy of the Bayesian network and neural network classifier was 81.8% and 90.2% respectively [29]. Based on the classical distribution model to simulate the training set and test set, the artificial neural network can get more than 95% classification effect [30]. Based on the analysis of the causes and characteristics of ionospheric clutter, the efficient classification and recognition of ionospheric radar clutter can be realized [31].

Clutter classification focuses mainly on sea clutter and meteorological clutter, but there are few studies on the classification of ground clutter. The premise of clutter classification is the extraction of various effective feature parameters. Our research found that compared with sea clutter and meteorological clutter, obvious differences occurred from UCA radar ground clutter, such as power spectrum characteristics, non-stationarity and nonlinearity, amplitude distribution characteristics, and correlation, etc. In addition, the design of the clutter classification algorithm is closely related to the characteristics of radar clutter and the specific application of radar. Therefore, the existing sea clutter and meteorological clutter classification methods are difficult to be directly applied to the UCA radar ground clutter classification. Particle Swarm Optimization (PSO) and Genetic Algorithm (GA) belong to Swarm Intelligence Optimization (SIO), so they can be used for reference in optimization and algorithm application. Applying genetic algorithm to the pattern recognition and classification of actual overvoltage data can realize effective data classification and recognition [32,33,34]. In addition, semi-supervised clustering based on the genetic algorithm can be used to effectively classify hyperspectral images [35]. Although GA can be well used in clustering classification, it has the problems of premature convergence and low local search efficiency. Introducing chaos theory into the standard PSO algorithm can effectively solve the problem of particle swarm easily falling into the local extreme point [36,37]. Therefore, in the field of unsupervised classification, GA clustering can be used to achieve data classification in different fields.

Based on the classification problem of clutter data collected by UCA ground-based radar, a data classification method based on the Chaotic Genetic Algorithm (CGA) is proposed in this paper. According to the ground clutter data collected in five different environments (microwave anechoic chamber, highway, dry grassland, town, and gravel land), three characteristics of the ground clutter data, including correlation, non-stationarity, and clutter spectrum (range-Doppler domain), were extracted, and the clutter data were classified by the analysis results of characteristic information. In addition, the optimization genetic algorithm of the chaos property is adopted to make the individuals move alternately between chaos and stability and the ergodic property of chaos theory is fully utilized to overcome the problem of precocious convergence of the population and improve the global convergence ability of the population.

In general, the main contributions of this paper are listed below:

- Firstly, the characteristics of ground clutter data measured in different UCA ground-based radar scenarios are studied, and the correlation, non-stationary, and statistical characteristics of the range-Doppler domain of clutter data are analyzed.
- Secondly, a GA clustering method based on chaos theory is proposed to overcome standard GA’s defects, such as premature convergence and weak local optimization ability, and complete the data classification and recognition according to the feature factors extracted from the measured clutter data.

The rest of this paper is organized as follows: In Section 2, the basic structure of the UCA radar system, the process of data preprocessing, the usual analysis method of ground clutter data (correlation analysis, non-stationary analysis, and statistical analysis of range-Doppler map), and the basic principle of clustering algorithm adopted in this paper are introduced. In Section 3, the classification results of ground clutter data in different scenarios and two kinds of beam control modes are presented and evaluate the two clustering algorithms’ classification performance. Finally, the conclusions and prospects of this study are summarized in Section 4.

## 2. Materials and Methods

### 2.1. Uniform Circular Array Radar and Experiment Sites

**Figure 1:**
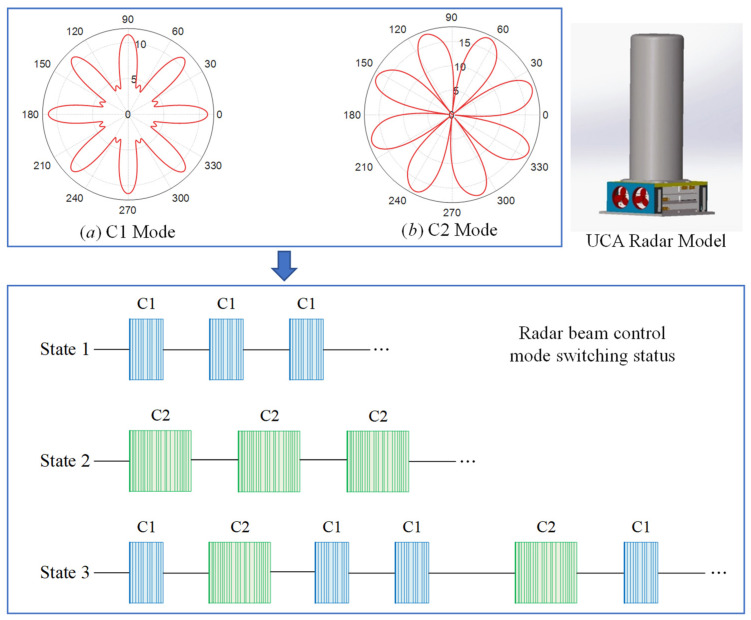
Switching states of two-beam control modes and corresponding beam pointing simulation pattern.

A uniform circular array radar is used for data collection. The radar system consists of a receiver, transmitter, transceiver antenna, signal processing unit, and power module. The antenna part comprises eight waveguide slot antennas with the same array element structure and adopts the operation mode of split transceiver. The UCA radar transceiver antenna is designed by eight conformal array elements with an interval of 45° along the circumferential azimuth direction, and the azimuth 360° full coverage circumferential scanning is realized by means of phase weighting. The antenna emission pattern is a multi-main-lobe pattern, and eight antennas receive signals independently. The simulation diagram of azimuth emission in two-beam control modes is presented in Figure 1. When the channels are in phase, the synthetic antenna’s emission pattern has eight main lobes, pointing to 0°, 45°, 90°, 135°, 180°, 225°, 270°, and 315° respectively (Figure 1a). When each channel is combined at a phase interval of 90°, the main lobe directions of the antenna emission pattern are 22.5°, 67.5°, 112.5°, 157.5°, 202.5°, 247.5°, 292.5°, and 337.5° respectively (Figure 1b). In addition, the pitching 3 dB beam width of a single element is 17.47°, and the pitching main beam direction of all transmitting and receiving array element is 90°. The radar system realizes the whole scene’s detection through an alternating transformation of the two beam-controlled modes. The beam control code switches to display three states during regular operation (Figure 1).

**Figure 2:**
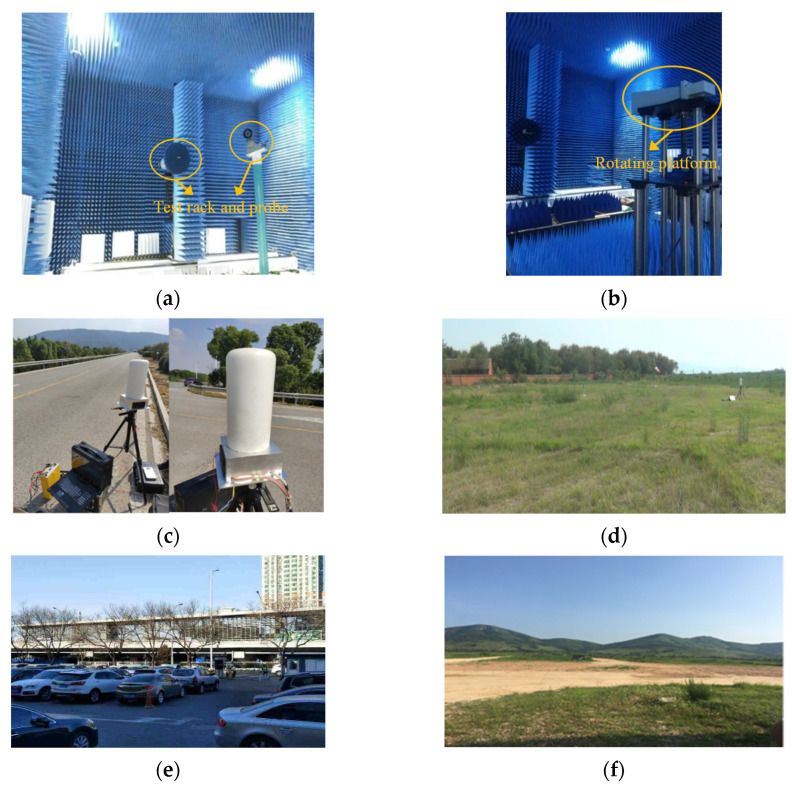
UCA radar data collection for different scenarios: (**a**) Microwave anechoic chamber test rack and probe; (**b**) Microwave anechoic chamber rotating platform; (**c**) Highway; (**d**) Dry grassland; (**e**) Town; (**f**) Gravel land.

The experiment includes microwave anechoic chamber experiment and field experiment (Figure 2). The microwave anechoic chamber is equipped with uniformly distributed absorbing materials and is equipped with a test stand and probe. Through the automatic control of the rotating platform, the radar system’s antenna parameters and the effectiveness of sending and receiving data can be easily tested (Figure 2b). Four scenarios with different environmental complexity were selected for multiple measurement experiments, which were located in Wuxi City, Jiangsu Province, Pinggu District, Beijing, Haidian District, Beijing, and Bengbu City, Anhui Province, China. According to topography, environment, and functional uses, it is classified as highway, dry grassland, town, and sandy land. Trees and shrubs are planted on both sides of the road with green protection belts (Figure 2c). There are a large number of weeds and a few low shrubs in the grassland monitoring area (Figure 2d). In the urban environment, a typical parking lot is selected, and there are several vehicles with strong interference parked in the monitoring area (Figure 2e). A little gravel and low weeds are scattered on the gravel ground (Figure 2f). Different background components and materials in the above selected experimental test scenes are the signal sources of ground clutter in the radar data under four different environments.

### 2.2. Data Pre-Processing

**Figure 3:**
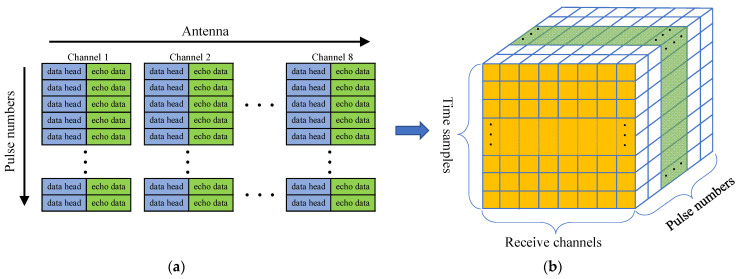
Radar data structure. (**a**) The storage format of echo data; (**b**) Radar data cube within a CPI.

Since each antenna of the radar receives signals independently, a pulse contains eight echo data channels, and the data structure is shown in Figure 3. In the distance dimension, the received signals are discharged in accordance with the sequence of channels and the pulse data of each channel contains frame header information, which is used to mark the pulse number and the wave-controlled mode (Figure 3a). Radar parameters such as pulse repetition frequency (PRF), sampling points of range dimension, and coherent processing interval (CPI) are slightly different among the data collected in different experimental scenarios. To observe the composition and structure of radar data, the frame header information is removed in Figure 3a, and multiple radar pulse data of eight receiving channels are spliced into a data cube (Figure 3b). The longitudinal direction represents the data sampling of the slow time dimension. The UCA radar data cube’s structure model is the basis for analyzing radar data characteristics and understanding signal processing operations.

**Figure 4:**
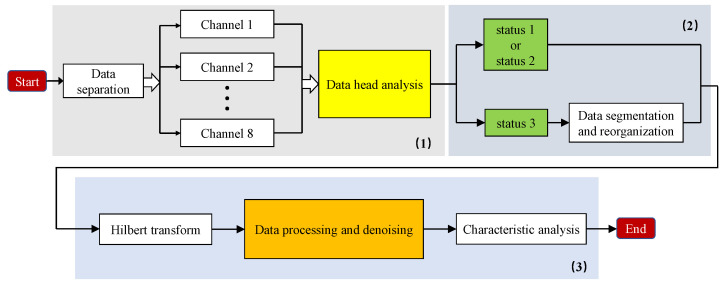
Data preprocessing flowchart.

Before analyzing the characteristics of ground clutter, it is necessary to preprocess the original data collected in different scenes, including three parts (Figure 4). Data processing steps of UCA radar are listed as follows: (1) Channel data separation: By analyzing the frame header information of eight channels’ pulse-echo data, the pulse sequence and beam control modes are marked; (2) Data extraction and reorganization determine the switching state of radar beam pointing, and data collected under the same beam control mode of eight channels are grouped into the same group; and (3) Data preprocessing and analysis, including Hilbert transform, range-direction FFT, denoising, normalization processing, and data statistical analysis.

Since the UCA radar’s working mode is the direct sampling of time-domain signals, it is necessary to perform a Hilbert transformation on the sampled signals and obtain two signals for coherent processing.

### 2.3. Characteristic Factors

The correlation, non-stationarity, and range-Doppler characteristics are selected as the characteristic factors of ground clutter and the characteristic differences in five different scenarios and two beam control modes are analyzed.

#### 2.3.1. Correlation Analysis of Ground Clutter Data

**Figure 5:**
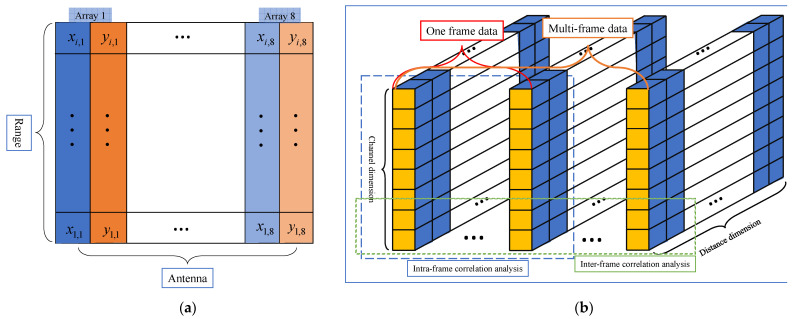
Schematic diagram of multi-dimensional data correlation analysis of UCA radar. (**a**) Schematic diagram of multi-channel single pulse data; (**b**) Schematic diagram of multi-channel and multi-pulse data.

Pearson correlation coefficient (PCC) is used to measure the correlation between different data types [38], and the beam-related correlation function, azimuth correlation function, and range-related correlation function are obtained. Beam correlation refers to the correlation between the sampled data obtained by two groups of different beam directions in the same channel, corresponding to the data samples represented by blue and brown in Figure 5a, one pulse has eight beam correlation coefficients. Azimuthal correlation refers to the correlation of clutter data of different beam directions in the same beam control mode, which corresponds to the data samples represented by the same color in Figure 5a. The range correlation is calculated from data samples of the same color in the same channel (single pulse sampling data of a beam). The case of multi-channel and multi-pulse data is shown in Figure 5b. Data samples with this structure can calculate short-term correlation (intra-frame data) and long-term correlation (inter-frame data). In addition, radar range-Doppler domain data can be calculated from single-frame data of one channel.

##### Radar Beam Correlation

Each channel of the UCA radar antenna can transmit or receive two beams indifferent directions, and the beam correlation is calculated by the data under different beam in the same channel. When extracting the characteristic factor of beam correlation, the data of different beams in the same channel in one frame are firstly taken to calculate the correlation (see Equation (1)) and the calculated results of eight channels acquired are averaged to obtain the finally calculated results for beam correlation of ground clutter data in one frame. The same pulse contains eight beam-correlation coefficients, so the beam-correlation function is shown as follow:

(1) $${\mathrm{BCF}}_{l}=\frac{\sum_{i=1}^{k}\left(x_{i,l}-{\hat{x}}_{l}\right)\left(y_{i,l}-{\hat{y}}_{l}\right)}{\sqrt{\sum_{i=1}^{k}{\left(x_{i,l}-{\hat{x}}_{l}\right)}^{2}\sum_{i=1}^{k}{\left(y_{i,l}-{\hat{y}}_{l}\right)}^{2}}},\begin{array}{cc} & l=1,2,\cdots ,8 \end{array}$$

where $x_{i,l}$ and $y_{i,l}$ are the distance unit sampling data of two beams in the same channel, ${\hat{x}}_{l}$ and ${\hat{y}}_{l}$ are the mean value of two groups of data, respectively. When extracting the beam correlation feature factor, firstly, the data of different beams in the same frame are taken to calculate the correlation, then the eight-channel calculation results are averaged to eliminate the influence of noise data. Finally, the beam correlation calculation results of one frame of ground clutter data can be obtained.

##### Azimuth Correlation

Azimuth correlation function refers to the range unit correlation of pulse data between different channels in the same beam control mode. The antenna beam direction is covered at equal intervals of 360°, realizing the whole scene range’s detection. Therefore, the azimuth correlation function can reflect the differences in different scenes, and it is defined as follows:

(2) $${\mathrm{AZCF}}_{j}=\frac{1}{\left(8-j\right)k}\sum_{l=1}^{8-j}\sum_{i=1}^{k}\left(x_{i,l}-{\hat{x}}_{l}\right)\left(x_{i,l+j}-{\hat{x}}_{l+j}\right),j=0,1,\cdots ,7$$

where $x_{i,l}$ and $x_{i,l+j}$ are the pulse distance unit data with two beam pointing intervals of *j* channels in the same beam control mode. The beam position interval of the same beam control mode covers the azimuthal direction of 360°, so the range unit data of adjacent spaced beams have a relatively strong correlation. Azimuth correlation depends not only on the beam position interval, but also on the wave control mode, and the latter has a more significant influence on the azimuth correlation. To distinguish the azimuthal correlation of two-beam control modes, the characteristic factor is obtained by averaging the correlation coefficients of different interval beam positions.

##### Range Correlation

The range correlation function of ground clutter is usually determined by the spatial difference of each range unit’s scattering distribution in the test scene. The correlation calculation formula between each distance unit is as follows:

(3) $${\mathrm{SPACF}}_{j}=\frac{1}{k-j}\sum_{i=1}^{k-j}\left(x_{i}-\hat{m}\right)\left(x_{i+j}-\hat{m}\right),j=0,1,\cdots ,k-1$$

where $x_{i}$ and $x_{i+j}$ are the radar sampling data in range direction with the interval of *j* between two range units, and $\hat{m}$ is the mean value of the sampling data of the range unit. The range-direction autocorrelation function gradually decreases with the increase of spatial distance sampling interval related to the beam control mode. When the sampling interval is 10, the average value of eight channels is calculated as the characteristic factor.

#### 2.3.2. Recursive Graph

A recurrence plot (RP) is used to analyze the non-stationary difference of ground clutter data under different beam control modes in the same scene of the UCA radar. Assuming the time series {x(1),x(2),⋯,x(N)}, a set of delay m-dimensional vectors can be obtained by the phase space reconstruction technique. The m-dimensional vector $\vec{X}\left(l\right)$ is as follows:

(4) $$\begin{array}{r} \vec{X}(l)={\left[x(l),x(l+\tau ),\cdots ,x(l+(m-1)\tau )\right]}^{T} \end{array},\begin{array}{cc} & l=1,2,\cdots ,L \end{array}$$

where $\vec{X}\left(l\right)$ is the delay vector, and the delay time is τ. At this time, L=N−(m−1)×τ is the number of reconstruction vectors, and the embedding dimension is *m*. Based on the embedding theorem [39], the appropriate phase space reconstruction parameters are selected for nonlinear time series. The Euclidean distance between any two points in L’s phase spaces is defined as:

(5) $$d_{i,j}=\left\|X\left(i\right)-X\left(j\right)\right\|,\begin{array}{cc} & i,j=1,2,\cdots ,L \end{array}$$

It can be seen from Equation (5) that X(i) and X(j) are any two points in the phase space, respectively. According to the Euclidean distance of each point in the phase space, the definition of the elements in the recursive graph can be obtained as follow:

(6) $$\begin{array}{c} R_{i,j}=\theta \left(\varepsilon -d_{i,j}\right),\begin{array}{cc} & i,j=1,2,\cdots ,N-\left(m-1\right)\tau \end{array}\\ \theta \left(\cdot \right)=\{\begin{array}{c} 1,\varepsilon -d_{i,j}>0\\ 0,\;\varepsilon -d_{i,j}<0 \end{array} \end{array}$$

where *i* is the number of rows, and *j* is the number of columns. The distance threshold is ε and θ(·) is the Heaviside function. When $d_{i,j}$ is less than the threshold ε, the value $R_{i,j}$ is 1. The recursion rate (RR) of the recursive graph of the UCA radar’s ground clutter sampling signal in range direction is used as the characteristic parameter to classify the beam control mode. RR refers to the ratio of the number of recursive points in a recursive graph to the total number of points. The calculation formula is:

(7) $$RR=\frac{1}{L^{2}}\sum_{i,j=1}^{L}R_{i,j}$$

The number of coordinate axis points in the recursive graph is *L* and $R_{i,j}$ is the current position’s recursive value. The larger RR is, the higher the recursion rate is. The RP describes the non-stationarity of the one-dimensional range-direction data, which is also related to the beam control mode. The average of the RR of the eight channels data is calculated as the characteristic factor.

#### 2.3.3. The Range-Doppler Maps

**Figure 6:**
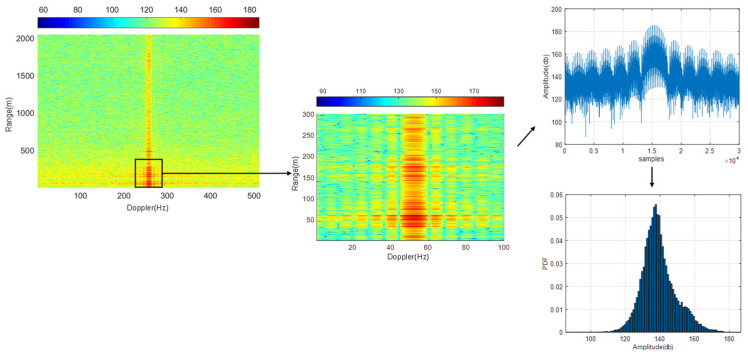
Schematic diagram of statistical parameter extraction of the range-Doppler map (Taking the dry grassland scene data as an example, 300 sample data are selected for the range unit, and 100 dimensions are chosen for the Doppler unit).

To analyze the statistical characteristics of the near-ground clutter region, the range-Doppler map is effectively clipped. Feature factors, including histogram mean and variance, are extracted from the clipped range-Doppler image’s statistical histogram. A frame of data includes range-Doppler data of eight channels in two-beam control modes. That is, it has a total of 16 range-Doppler images. The final feature factor of a frame of data is formed by taking the average of these images’ histogram statistical results. Figure 6 illustrates the process of range-Doppler clipping and statistical analysis by taking the dry grassland scene as an example. By extracting the clipping region’s sample data, the statistical histogram is made, and the mean value and variance of the sample data are calculated. It can be obtained as follows:

(8) $$\{\begin{array}{l} \begin{array}{l} \bar{c}=\frac{\sum_{m=1}^{M}\sum_{n=1}^{N}c_{mn}}{MN} \end{array}\\ \sigma =\sqrt{\frac{1}{MN-1}\sum_{m=1}^{M}\sum_{n=1}^{N}{\left(c_{mn}-\bar{c}\right)}^{2}} \end{array}$$

where $c_{mn}$ is the amplitude value at positions *m* and *n* in the range-Doppler map, and $\bar{c}$ represents the mean value of clutter amplitude in the clipped region, where the number of distance units is *M*, and the number of Doppler units is *N*. One frame of data includes a total of 16 range-Doppler images of eight channels in two-beam control modes. Based on the mean value of statistical histogram results, the range-Doppler domain feature factor of frame data is formed.

##### Feature Factor Extraction and Analysis

**Figure A1:**
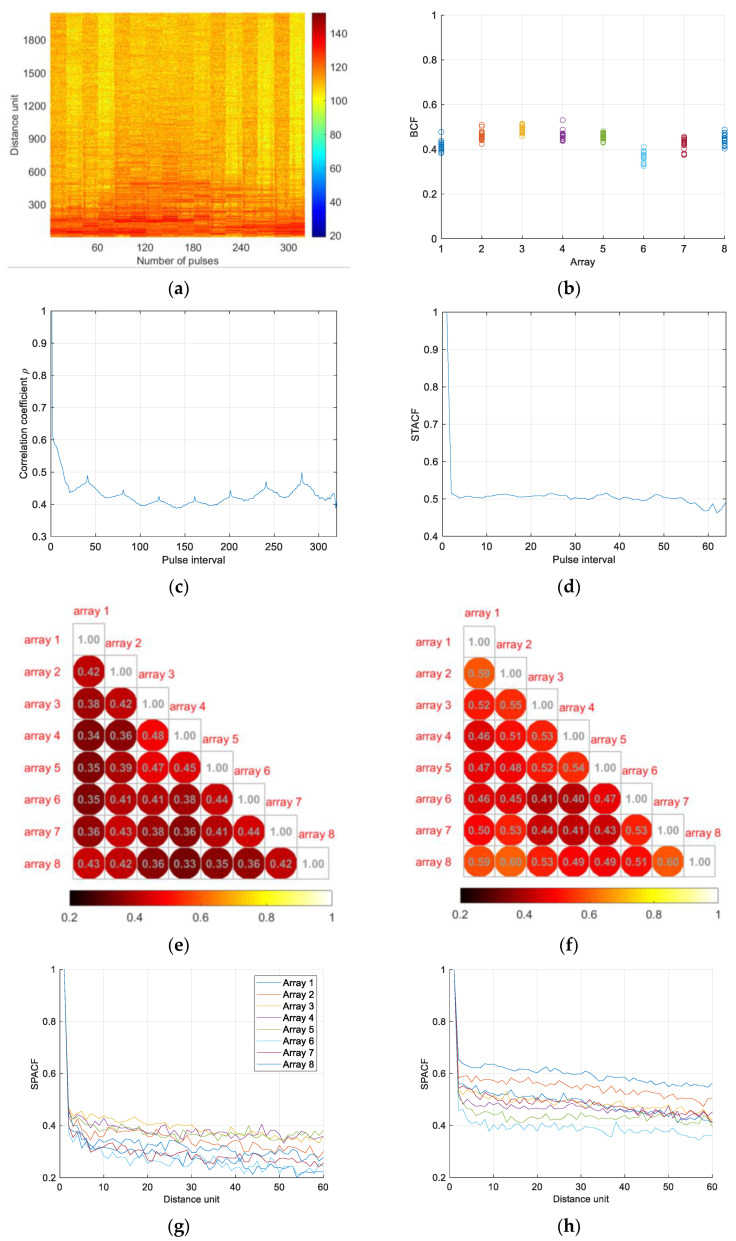
Correlation characteristic map of UCA radar ground clutter data. (**a**) Range time diagram of full-channel pulse splicing data; (**b**) Beam correlation diagram of eight channels of radar; (**c**) The variation diagram of inter pulse correlation coefficient corresponding to (**a**); (**d**) Intra-frame correlation function diagram of C1 beam for channel 1; (**e**) Azimuth correlation matrix diagram of C1 beam; (**f**) Azimuth correlation matrix diagram of C2 beam; (**g**) Range autocorrelation of eight channels of the C1 beam; (**h**) Range autocorrelation of eight channels of the C2 beam.

**Figure A2:**
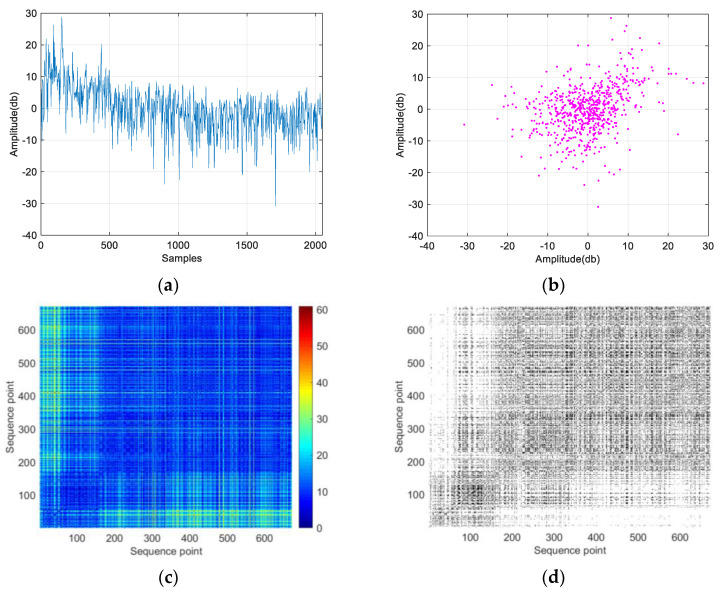
Recursive analysis results of range sampling signal of UCA radar ground clutter. The data comes from the UCA radar test experiment in the dry grassland scene. The scene’s clutter background is relatively clean, and the change of the ground structure in each distance unit within the irradiation range of the beam in the fifth channel is noticeable. (**a**) One dimensional sampling signal in range direction; (**b**) Phase space plan; (**c**) State distance matrix; (**d**) Recursive plot.

Correlation characteristic map and recursive analysis results of range sampling signal of UCA radar ground clutter can be seen from Figure A1 and Figure A2. Three different characteristic factors of radar beam correlation, range-Doppler domain mean, and variance are selected to cluster the ground clutter data of different scenes. Three characteristic factors of the azimuth correlation function, range correlation coefficient, and recursive rate are selected to cluster the ground clutter data of different beam control modes in the same scene (Table 1, takes the ground clutter data obtained from the Wuxi highway scene as an example to calculate the value range of six characteristic factors). The UCA radar has eight channels and 2048 range sampling units. Reading five frames of data, each frame of data pulse number is 64, so a total of 320 pulse samples. Two different beam control modes are distinguished, and the sample size is 640.

### 2.4. Clustering Algorithms

Aiming at the defects of standard genetic algorithm, an improved genetic algorithm is proposed to realize the clutter data clustering function by introducing chaotic sequences’ characteristics.

#### 2.4.1. Standard Genetic Algorithm Clustering

The standard GA algorithm constructs the fitness function by searching the minimum clustering center of the objective function value:

(9) $$F=\frac{1}{\sum_{j=1}^{c}\sum_{k=1}^{n_{j}}{\left\|x_{k}^{\left(j\right)}-m_{j}\right\|}^{2}},m_{j}\left(j=1,2,\cdots ,c\right)$$

where $x_{k}$ is the sample and the clustering as center is $m_{j}$. Firstly, a population is initialized randomly, and the clustering center is encoded by floating point number. The coding of the clustering center is optimized through selection, crossover, and mutation operations, then the result of clustering division is judged based on the sum of eigenvectors of samples to be classified and the corresponding Euclidean distance of the clustering center. Floating-point coding is to regard a chromosome as a string composed of K clustering as centers, that is, the K clustering analysis of D-dimensional feature vectors and the chromosome structure of the clustering center is defined as:

(10) $$f=\left\{x_{11},x_{12},\cdots ,x_{1d},x_{21},x_{22},\cdots x_{2d},\cdots ,x_{k1},x_{k2},\cdots ,x_{kd}\right\}$$

where the feature dimension of the data sample is *d*, and *k* represents the central cluster, that is, each chromosome is a floating-point code string with the length of k×d.

#### 2.4.2. Chaotic Genetic Algorithm Clustering

Based on the standard genetic algorithm, we propose an improved idea, which introduces chaotic disturbance terms at the initial position and the local convergence position respectively, which is called chaotic genetic algorithm in this paper. The ergodicity of chaos theory can overcome local minima and achieve global optimization. Adding a chaotic sequence when the population is initialized can give full play to the global optimization ability of the genetic algorithm, and improve the convergence speed of the population by using the ergodicity of chaotic particles. The introduction of chaotic disturbance term at the local convergence position can break the monopoly position of individual dominant individuals in the population, and prevent the group from losing its competitiveness, which not leading to the stagnation of the population.

**Figure 7:**
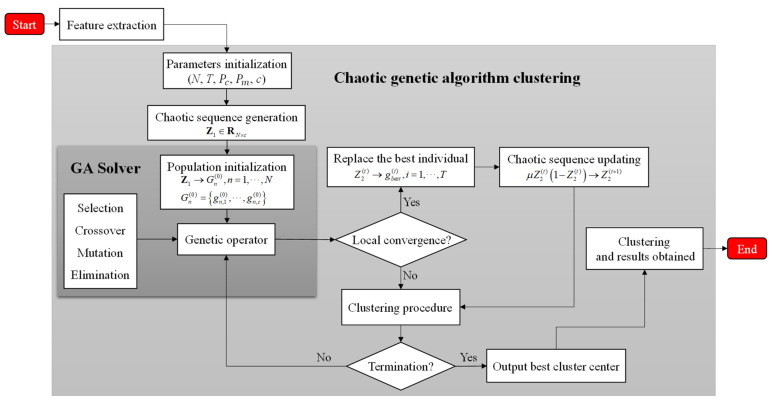
Chaotic genetic algorithm clustering flowchart.

At the initial position and local convergence position of the standard GA, the logistics equation is adopted to introduce the interference term to generate chaotic sequences, which are determined by the following equation:

(11) $$z^{\left(t+1\right)}=\mu z^{\left(t\right)}\left(1-z^{\left(t\right)}\right),t=0,1,2,\cdots$$

where $z^{\left(0\right)}\in \left(0,1\right)$ and $z^{\left(0\right)}\notin \left(0.25,0.5,0.75\right)$, when μ=4, the generated sequence is completely chaotic. The population size (labeled as N) is set to be within 100 and the maximum number of iterations (labeled as T) to be 1000. The evolutionary process of the population is ended by judging the termination conditions. The hybridization probability $P_{c}$ and mutation probability $P_{m}$ are 0.9 and 0.01, respectively. The GA based on chaos theory is obtained by combining chaos theory with GA, and its process is shown in Figure 7.

In the initial stage, the chaotic sequence corresponding to the population size is generated by a chaotic operator to initialize the population. The individual elimination in the process of GA Solver adopts the method of survival of the fittest, that is, the dominant individuals replace the poorest individuals. Chaos genetic algorithm involves selection, crossover and mutation operations, respectively, using roulette algorithm, single-point crossover algorithm, and basic bit algorithm. There are two termination conditions of the algorithm, one is to achieve the set number of iterations, the other is the clustering criterion function to achieve the set threshold. In the process of population evolution, the quantitative calculation is introduced to judge whether the population is precocious, as shown in the following formula:

(12) $$\begin{array}{c} \lambda_{s}^{2}={\sum_{i=1}^{n}\left(\frac{F_{i}-F_{avg}}{F}\right)}^{2}\\ F=\max\left\{1,\max\left[\left|F_{i}-F_{avg}\right|\right]\right\},\;i=1,2,\cdots ,n \end{array}$$

where $\lambda_{s}^{2}$ is the population fitness variance of the population, representing the aggregation degree of individuals of the population. When $\lambda_{s}^{2}<C$ (C is a constant), the population falls into precocity. $F_{i}$ is the fitness value of the current iteration and $F_{avg}$ is the current average fitness of the population. F is the normalization factor.

## 3. Results

This section mainly introduces the clustering results of UCA radar clutter measured data and uses the criterion function based on Euclidean distance to evaluate GA clustering’s performance and chaotic GA clustering proposed in this paper. The feature factor is normalized according to the maximum and minimum value of the feature factor. In the scene data clustering analysis, five clustering centers are set according to different scenes of measured data. In the clustering analysis of beam control mode data, two clustering centers are set. 

### 3.1. Clustering of Clutter Data in Different Scene

The analysis results come from five experimental scenes, and the population size is set to 10, 15, 20, 25, and 30, and the maximum number of iterations is set to 1000. The process of population evolution is terminated when the central cluster reaches the specified minimum Euclidean distance (the value is actually 20) or exceeds the maximum number of iterations. The clustering experiment in each case is performed five times, and the clustering result is the average value of five times (Table 2). Compared with SGA clustering, the chaotic SGA clustering proposed in this paper has obvious advantages, which are mainly reflected in two aspects:

1. It has a faster convergence speed, which can save 34.60% of the time.
2. It has a higher classification accuracy, and the average criterion function value is reduced by 42.82%.

As the population size increases, the classification accuracy of both SGA and chaotic SGA both show an upward trend (from 20% and 40% to 60% and 100%, respectively), while the criterion function decreases significantly (from 49.52 and 23.63 to 28.59 and 21.03, respectively), and the convergence speed of the population gradually slows down (the average convergence speed of SGA clustering increased from 29.85 to 80.31, while chaotic SGA clustering increased from 23.63 to 43.31). Since the introduction of chaotic interference SGA clustering can shorten the time for the population to jump out of the local optimal position, the convergence speed of the chaotic SGA decreases slowly. Besides, the average evolution times of SGA clusters have reached or approached the set maximum evolution generation of 1000, which means that the population is at risk of falling into a local optimum.

From the cluster analysis of the population size, we can see that the larger the population size and evolutionary generation, the better the clustering effect, but the slower the convergence speed. Based on the two-way trade-off between classification accuracy and convergence speed, the population size is set to 20 in the experimental analysis of this study.

**Figure 8:**
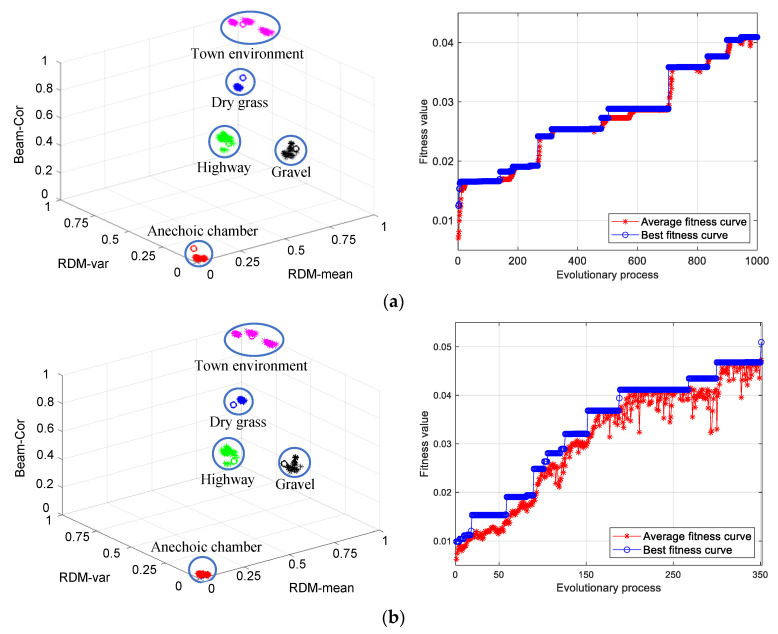
Clustering results of SGA (**a**) and chaotic SGA (**b**) in different scenarios. The data under five different experimental scenarios are selected, the population size is set to 20, the maximum number of iterations is set to 1000, and the termination condition of the minimum criterion function value is 20. In the figure on the left, the star-shaped points are the data sets, and the hollow circles are the cluster centers of the corresponding data. Note. The variables represented by the axis in the figure are dimensionless.

When the population size of SGA clustering and chaotic SGA clustering is set to 20, the clustering results and the evolution process of corresponding populations under five different experimental scenes are shown in Figure 8. Compared with SGA clustering, the clustering center obtained by chaotic SGA clustering is more consistent with the classification and division of actual characteristic factors, and the corresponding criterion function values are 24.27 and 19.63, respectively. At the same time, it can be seen from the iterative process of the two algorithms that the average fitness curve and the optimal fitness curve of the SGA clustering evolution process are in the state of fitting for a long time. From the evolution process graph of chaotic SGA clustering, it can be seen that the average fitness function curve shows an upward trend after chaotic disturbances are introduced at the initial position and the local optimal position, respectively. Besides, compared with SGA clustering (55.51 s), chaotic SGA clustering takes less time to find the clustering center (21.04 s).

### 3.2. Clutter Data Clustering of Two-Beam Control Modes in The Same Scene

Taking the highway scene as an example, the clustering analysis results of ground clutter data with different beam control modes are presented in Table 3. The population size is set as 5, 10, 15, 20, and 25, and the maximum number of iterations is set as 500. When the cluster center reaches the specified minimum Euclidean distance (which is actually 75) or exceeds the maximum number of iterations, the population evolution process is terminated. The experiments are repeated five times for each population size, and the clustering performance is evaluated by taking the mean value of the five results.

As can be seen from Table 3, compared with SGA clustering, the chaotic SGA clustering process has a faster convergence speed and smaller criterion function value, with the convergence speed increased by 42.69% and criterion function decreased by 20.79%, while the number of evolutions is less (decreased by 41.69%). The results of several experiments show that the classification effect is good only when the value of the criterion function is lower than 75, while the SGA clustering algorithm is greater than this value even when the population size is 25. Therefore, the SGA clustering algorithm cannot well realize the classification of the two-beam control modes’ data. Besides, with the increase of population size from 10 to 25, the average value of criterion function of chaotic SGA is between 73 and 74, and the classification accuracy does not significantly improve with the increase of the population size. However, the number of iterations decreased significantly from 340 to 238. Compared with SGA clustering, chaotic SGA clustering shows a better superiority in both local and global search capabilities.

**Figure 9:**
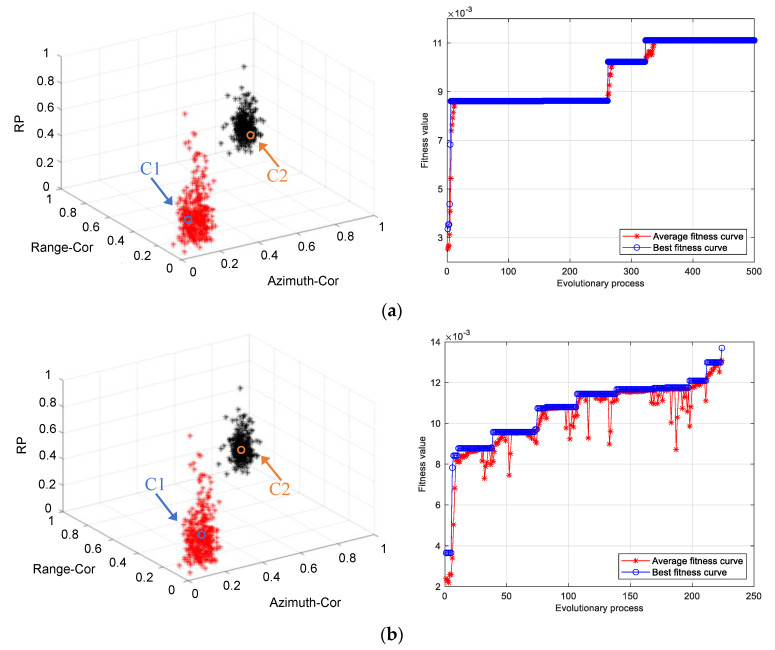
Clustering results of SGA (**a**) and chaotic SGA (**b**) in two-beam control modes. The population size is set to 15, and the maximum number of iterations is set to 500. The termination condition of the minimum criterion function value is 75. Note. The variables represented by the axis in the figure are dimensionless.

When the population size is greater than 10, chaotic SGA clustering can realize the effective classification of data under two beam control modes. Set the population size as 15, and the clustering results of SGA and chaotic SGA are shown in Figure 9. The average fitness curve of chaotic SGA increased steadily, and due to the influence of the retraction of the average fitness curve, the stagnation period of the optimal fitness curve in the evolutionary process diagram is relatively short, and the population jumps out of the optimal local position and has relatively strong local search ability (the right of Figure 9b). Although both SGA and chaotic SGA clustering can obtain classification results that match the actual beam control mode, the criterion function values of the clustering center are obviously different (SGA is 90.02, and chaotic SGA is 72.99). At the same time, compared with SGA clustering (21.5373 s, 500), the convergence speed of the chaotic SGA clustering process is faster (12.71 s), and the number of evolution generation is smaller (223). Therefore, chaotic SGA clustering uses a shorter evolutionary generation than SGA clustering to complete the global optimal position search, which has stronger global searchability.

## 4. Conclusions

The ground clutter data collected in five field test environments of UCA radar are analyzed, and the effective classification of data in different scenes and two types of beam control modes is completed. In this paper, statistical characteristics (mean value, variance) of the range-Doppler domain and beam correlation feature factors are extracted as scene classification of clutter data. In addition, the azimuth correlation, range autocorrelation, and recursive plot recursive feature factors are extracted as beam control mode classification of clutter data. Finally, the chaotic genetic algorithm proposed in this paper is used for data clustering analysis under five different scenes and two different beam control modes. The conclusions are as follows:

1. Compared with SGA clustering, the clustering center obtained by chaotic SGA clustering is more consistent with the classification and division of actual characteristic factors. From the scene data, the criterion function values of SGA and chaotic SGA clustering corresponding to scene classification are 38.03 and 21.74, respectively, and the time consumed is 55.57 and 36.34 s, respectively. From the beam control mode of data classification, the criterion functions are 93.99 and 74.45, respectively, and the convergence speeds are 17.47 and 10.01 s, respectively.
2. Chaotic SGA clustering has high local search ability and global searchability, realizing the effective classification of data samples.
3. The effective classification and analysis of ground clutter data can improve UCA radar adaptability to clutter environments to enhance target detection performance.

In the future, more ground clutter data of measurement experiments will be carried out to improve the establishment of a ground clutter database in different scenes. It realizes the subtle perception of ground clutter characteristics of UCA radar and provides more space modeling information of ground clutter for the application of auxiliary adaptive detection technology.

## Figures and Tables

**Table 1:** Feature factor for clustering process of different scenarios and beam control modes.

Feature Factors	Minimum	Maximum	Samples	Categories	Cluster Experiment
RDM-mean	119.3997	139.1942	320	5	Scene classification
RDM-variance	55.9213	166.1000			
Radar beam correlation	0.0973	0.6299			
Azimuth correlation	0.3490	0.5171	640	5	Beam steering mode classification
Range correlation	0.1528	0.4497			
Recursive rate of recursive graph	0.1693	0.5601			

Note. The values of all characteristic factors are normalized in the process of clustering.

**Table 2:** Performance evaluation of SGA and chaotic SGA clustering under different population sizes.

Algorithm	Population Size	Number of Runs	Average Evolutionary Generation	Average of Convergence Rate (s)	Mean Value of Criterion Function	Classification Accuracy
SGA Clustering	10	5	1000	29.85	49.52	20%
	15	5	1000	43.18	46.27	40%
	20	5	959	54.52	35.23	40%
	25	5	1000	69.99	30.53	60%
	30	5	988	80.31	28.59	60%
Chaotic SGA Clustering	10	5	933	28.14	23.63	40%
	15	5	827	35.00	23.11	60%
	20	5	643	35.72	20.65	80%
	25	5	586	39.53	20.30	100%
	30	5	530	43.31	21.03	100%

**Table 3:** Performance evaluation of SGA clustering and chaotic SGA clustering under two beam control modes (data from highway scene experiment).

Algorithm	Population Size	Number of Runs	Average Evolutionary Generation	Average of Convergence Rate (s)	Mean Value of Criterion Function
SGA Clustering	5	10	500	7.76	132.24
	10	10	500	12.45	89.94
	15	10	500	17.60	86.76
	20	10	472	21.95	81.52
	25	10	475	27.57	79.49
Chaotic SGA Clustering	5	10	340	4.91	77.82
	10	10	281	7.29	73.42
	15	10	293	11.00	74.09
	20	10	275	13.00	73.69
	25	10	238	13.87	73.20

## Data Availability

The data presented in this study are available on request from the corresponding author. The data are not publicly available due to privacy.
